# Thermodynamic metabolic modeling of growth and bioproduction potential of the acetogen *Acetobacterium woodii* with and without redox cofactor swaps

**DOI:** 10.1016/j.synbio.2026.05.007

**Published:** 2026-06-13

**Authors:** Jasmin Bauer, Axel von Kamp, Stefan Pflügl, Steffen Klamt

**Affiliations:** a Max Planck Institute for Dynamics of Complex Technical Systems, Sandtorstr. 1, Magdeburg, Germany; b Institute for Chemical, Environmental and Bioscience Engineering, Technische Universität Wien, Gumpendorfer Straße 1a, Vienna, 1060, Austria

**Keywords:** Constraint-based metabolic model, Redox metabolism, C1 metabolism, Systems biology of acetogens, Metabolic engineering, Thermodynamics, Max-min driving force

## Abstract

Acetogenic bacteria such as *Acetobacterium woodii* use the Wood-Ljungdahl pathway to convert H_2_/CO_2_ and other C1 substrates into acetate and couple it via chemiosmotic energy conservation with ATP synthesis. To coordinate the associated electron flows under tight thermodynamic constraints, acetogens use not only NAD(H) and NAD(P)H but also ferredoxin as a third major redox cofactor. In this work, we systematically explore how cofactor specificity in redox reactions, including potential cofactor swaps, may influence growth and bioproduction.

We initially reconstructed and validated a large-scale constraint-based metabolic model of *A. woodii* equipped with standard Gibbs-free-energy values and metabolite concentration bounds.

We then analyzed the effects of swapping redox cofactors in the model. This analysis revealed that, in theory, suitable cofactor swaps could increase the growth rate by a factor of 5 compared to the wild type, but only under very high H_2_ and CO_2_ concentrations and with tight ranges for the redox states of the cofactors. More realistic solutions with higher driving forces and broader concentration ranges would still enable an up to 2.5-fold increase in growth rate and are based on two key swaps: (a) replacement of the hydrogen-dependent CO_2_ reductase (HDCR) by a NADPH-dependent formate dehydrogenase and (b) substitution of NAD^+^ by NADP^+^ in the bifurcating hydrogenase. These variants, which were frequently favored by the algorithm in different scenarios and are used by other acetogens, increase the amount of reduced ferredoxin available for subsequent ATP generation. Unexpectedly, our analysis further revealed that the use of ferredoxin and NADH alone (in combination with suitable cofactor swaps) could lead to similar growth rates and driving forces as for the wild type. We discuss possible reasons why these solutions may not have been selected by evolution. In particular, we show that the native redox cofactor specificities of *A. woodii* facilitate near-maximal driving forces under a wide range of H_2_ and CO_2_ concentrations.

Finally, we evaluated production of 15 native and heterologous chemicals from four C1-substrate regimes. This analysis reveals that targeted cofactor engineering can in many (but not all) cases (i) enable growth-coupled synthesis of a target chemical if it is infeasible in the native *A. woodii* strain or (ii) enhance the thermodynamic driving force of product synthesis.

Overall, this work provides a valuable resource and a generalizable thermodynamics-based framework for evaluating redox engineering strategies in acetogens and other energy-limited microorganisms.

## 1 Introduction

Acetogens are a diverse group of obligate anaerobic microorganisms that use the Wood-Ljungdahl pathway (WLP) for both assimilation and energy conservation (ATP synthesis) with acetate as primary fermentation product [[1], [2], [3], [4], [5]]. Their ability to convert H_2_ and CO_2_ (as well as syngas and other C1-substrates) to organic compounds has recently gained high interest in the context of sustainable bioproduction processes [[6], [7], [8], [9], [10], [11], [12], [13], [14], [15], [16], [17], [18]], and some first industrial applications have already been established [11]. *Acetobacterium woodii* is one of the earliest discovered and most investigated acetogens [1,19,20]. It is considered as a promising candidate for sustainable bioprocesses [6,17,21]. In their natural habitat, the assimilation of hydrogen (H_2_) and CO_2_ via the WLP is of particular importance, however, a variety of other substrates, including CO [[21], [22], [23]], methanol [24,25] and formate [23,26,27], can be metabolized by *A. woodii* as well. There is a growing number of studies addressing metabolic engineering of *A. woodii* for sustainable production of chemicals [14,23,[28], [29], [30], [31], [32], [33]] and quantitative approaches have been used to analyze its industrial potential [21,26].

Despite the increasing attention towards acetogens, computational metabolic models, which have been an invaluable tool to study and engineer the metabolism of many other microorganisms [[34], [35], [36], [37]], are still relatively scarce for acetogens. The potential of metabolic models in advancing our understanding of acetogen metabolism, especially regarding thermodynamic and energetic properties, has been highlighted by Heffernan et al. [38], who also provided an overview of existing models for acetogens. While genome-scale constraint-based (stoichiometric) metabolic models have been published for selected acetogenic species (e.g., *Clostridium ljungdahlii* [39], *Clostridium autoethanogenum* [40,41], and *Moorella thermoacetica* [42]), we could not find a curated large-scale metabolic model for *A. woodii* in the peer-reviewed literature. A larger stoichiometric model of *A. woodii* was mentioned in a conference abstract by Mesfin et al. [43] and in a related doctoral thesis [44], but the model itself or any code of implementation was not provided with these studies. Smaller metabolic models of *A. woodii* have been published and used in Refs. [21,26,45], however, these models are neither charge- nor mass-balanced and cover only central metabolic pathways with a simplified biomass synthesis reaction [26].

One particular aspect of the metabolism of acetogens is the thermodynamics of the WLP and associated electron flows, which are often at the edge of thermodynamic feasibility, and how the cells achieve redox balance. In contrast to many other organisms, which use NAD(H) and NAD(P)H as universal redox cofactors in their metabolism, acetogens possess, in addition, ferredoxin as redox carrier giving them an extra degree of freedom to coordinate electron flows and to enable net ATP formation along the WLP pathway. It turns out that central redox reactions may differ in acetogenic species regarding the used redox cofactors, especially in the WLP. This raises the question which redox cofactor specificities might be optimal in terms of thermodynamic driving force or, in the context of metabolic engineering, which cofactor swaps may be beneficial for the production of a certain target chemical. Computational studies on (optimal) cofactor specificities and swaps have been conducted with *Saccharomyces cerevisiae* [46,47] and *E. coli* [[47], [48], [49], [50], [51]] in the context of metabolic engineering. Recently, we introduced the TCOSA method (Thermodynamics-based COfactor Swapping Analysis) and used it to analyze the optimality of the NAD(P)(H) specificity of redox reactions in *E. coli* with respect to network-wide thermodynamic driving forces [52].

In this work, we first built an extended charge- and mass-balanced constraint-based metabolic model of *A*. *woodii* endowed with thermodynamic parameters. After initial (thermodynamic) flux balance analyses that demonstrated the validity of the model, we used different constraint-based modeling techniques, including TCOSA, (a) to analyze the optimality (and potential variants) of redox cofactor specificities in *A. woodii* for growth and (b) to assess the production capabilities of *A. woodii* for a range of relevant substrates and products, both with and without cofactor swaps.

## 2 Methods

### 2.1 Thermodynamic flux balance analysis and MDF calculations

#### 2.1.1 Optimization problems

For (thermodynamic) flux balance analysis and calculations related to the max-min driving force (MDF), we consider herein a constrained-based metabolic network model that integrates steady-state mass balances for the metabolites (eq. (1)), bounds on metabolic fluxes (eq. (2)), and five additional inequalities ensuring that the thermodynamic driving forces of the active reactions do not fall below a given threshold $f^{\min}$ (eqs. (3)–(7)):

(1) N·r=0

(2) $$\alpha_{i}\leq r_{i}\leq z_{i}\cdot \beta_{i}$$

(3) $$f_{i}=-\Delta G_{i}^{\prime }=-\Delta G_{i}^{\prime }{}^{\circ}-RT\cdot {\left({\hat{N}}_{\cdot ,i}\right)}^{T}\cdot x$$

(4) $$\ln\left(c_{i}^{\min}\right)\leq x_{i}\leq \ln\left(c_{i}^{\max}\right)$$

(5) $$B\leq f_{i}+M\cdot \left(1-z_{i}\right)$$

(6) $$B\geq f^{\min}$$

(7) $$z_{i}\in \left\{0,1\right\}$$

N is the stoichiometric matrix containing the stoichiometry of all reactions ($N_{j,i}$ stores the: stoichiometric coefficient of the intracellular (internal) metabolite j in reaction i). Each rate $r_{i}$ has a lower ($\alpha_{i}$) and an upper ($\beta_{i}$) bound. Herein we assume that, in calculations, all reversible reactions are split into two irreversible reactions (hence, $\alpha_{i}\geq 0$ for all reactions). For the $r_{ATPM}$ reaction quantifying non-growth associated ATP consumption we use a lower bound of 0.29 mmol·g_DW_^−1^·h^−1^ previously reported for *A. woodii* [45]. $\Delta G_{i}^{\prime }{}^{\circ}$ is the standard Gibbs free energy of reaction i, R the universal gas constant, T the temperature (here: 298.15 K). The driving force $f_{i}$ of reaction i is defined as the negated Gibbs free energy change ($\Delta G_{i}^{\prime }$) of this reaction. $\hat{N}$ is the extended stoichiometric matrix, which includes in its rows both internal and external metabolites. ${\hat{N}}_{\cdot ,i}$ is the i-th column (reaction) of the extended stoichiometric matrix and x is the vector of the logarithmic concentrations of the metabolites. The latter are also restricted by lower and upper bounds. The variable B represents a lower bound of the thermodynamic diving forces of all active reactions, M is a number greater than the maximal possible driving force in the network, and $z_{i}$ is a binary variable that must be 1 if reaction i is active ($r_{i}\;>0$).

The constraints (1)–(7) provide the frame for different optimization problems used herein:

• *Flux Balance Analysis (FBA)*: Maximization of a specific reaction rate $r_{obj}$, typically the growth rate (μ) or the production rate of a certain compound $(r_{P}$), subject to the basic constraints (1)–(2) (for FBA, the $z_{i}$ are set to 1 in eq. (2)). This is a simple linear optimization problem (linear program, LP).
• *Thermodynamic Flux Balance Analysis (TFBA*): Maximization of a specific reaction rate $r_{obj}$ subject to stoichiometric and thermodynamic constraints (1)–(7). Herein we demand a minimum driving force ($f^{\min}$) of 0.1 kJ mol^−1^. Due to the binary variables $z_{i}$, this is a mixed-integer linear program (MILP).
• *Max-min driving force (MDF)*: searches for a flux distribution that maximizes the smallest driving force of all active reactions (represented by variable B in eqs. (5) and (6)), subject to the constraints (1)–(7). Sometimes, certain fluxes (e.g. the growth rate) are fixed in these calculations to find the MDF (and the associated flux distribution) for this particular scenario. This optimization problem is also a MILP and corresponds to the OptMDF approach presented in Ref. [53].

#### 2.1.2 Calculation of standard Gibbs free energies

We used the eQuilibrator API [54] to calculate the standard Gibbs free energies $\Delta_{r}G^{\prime }{}^{\circ}$ needed for the TFBA and MDF optimizations. The cytosolic pH was set to 7.5 and an ionic strength of 0.25 M as well as a pMg of 3.0 were assumed. A specific consideration was necessary in the case of ferredoxin. The eQuilibrator database only allows for a singly negatively charged form of ferredoxin (transfer of one electron), whereas acetogenic bacteria commonly use the doubly negatively charged form (transfer of two electrons: Fdx_ox_ + 2 e^−^ → Fdx_red_). The standard Gibbs free energy change of redox reactions with participation of ferredoxin is therefore calculated via the standard redox potentials of the respective half-reactions. For example, the bifurcating hydrogenase of *A. woodii* catalyzes the reaction 2H_2_ + NAD^+^ + Fdx_ox_ → 3 H^+^ + NADH + Fdx_red_. The half-reactions are Fdx_ox_ + 2 e^−^ → Fdx_red_ (acceptor reaction) and 2 H_2_ + NAD^+^ → 3 H^+^ + NADH + 2 e^−^ (cumulated donor reaction). For the first half-reaction we use a standard redox potential of $E_{{Fdx}_{ox}\to {Fdx}_{red}}^{\prime {}^{\circ}}=-450\;\text{mV}$ for ferredoxin [1]. For the second half reaction we use the standard redox potential of −743mV delivered by eQuilibrator. The standard redox potential difference of the entire reaction is then ${\Delta E}^{\prime }{}^{\circ}=$ $E_{acceptor}^{\prime {}^{\circ}}-E_{donor}^{\prime {}^{\circ}}$ = −450mV−(−743mV)=293mV. Using the Nernst equation

$$\Delta G^{\prime }{}^{\circ}=-n\cdot F\cdot {\Delta E}^{\prime }{}^{\circ}\text{,}$$

with n=2 transferred electrons and Faraday constant F, we obtain a $\Delta G^{\prime }{}^{\circ}$ of −56.59 kJ mol^−1^. It is important to note that this value is significantly more negative than other reported $\Delta G^{\prime }{}^{\circ}$ values for the bifurcating hydrogenase reaction (e.g. −11 kJ mol^−1^ in Ref. [1]). This discrepancy arises because eQuilibrator utilizes a standard redox potential of $E^{\prime }{}^{\circ}=-543$ mV for H_2_ (rather than the conventional −414 mV) corresponding to a standard state of 1 M dissolved H_2_, whereas the conventional value is based on a partial pressure of 1 bar gaseous H_2_. This approach is necessary for consistency, as our calculations of Gibbs free energy changes are performed with respect to molar concentrations. However, given the typically very low concentrations of dissolved H_2_ (see also below) and the fact that the ferredoxin pool in the cell is predominantely in the reduced state, the actual in vivo $\Delta G^{\prime }$ of the hydrogenase reaction is expected to be much less negative (closer to equilibrium).

Two reactions involve translocation of Na^+^ ions over the cytosolic membrane: the Na^+^-pumping ferredoxin:NAD oxidoreductase complex (RNF complex; ${\text{Fdx}}_{\text{red}}+H^{+}+2\;{\text{Na}}_{\text{cyto}}^{+}+{\text{NAD}}^{+}\;\to \;{\text{Fdx}}_{\text{ox}}+2\;{\text{Na}}_{\text{peri}}^{+}+\text{NADH}$) and the Na^+^-dependent ATP synthase ($\text{ADP}+H^{+}+3.3\;{\text{Na}}_{\text{peri}}^{+}+P_{i}\;\to \;\text{ATP}+H_{2}O+3.3\;{\text{Na}}_{\text{cyto}}^{+}$). For those reactions we have to add an energy term ($\Delta G_{Na+}^{\prime {}^{\circ}}$) to the standard Gibbs free energy change of the reaction to account that the transport takes place along a Na^+^-motive force (here formulated in the direction from higher (periplasmic space) to lower (cytosol) Na^+^ concentration [[54], [55], [56]]:

$$\Delta G_{Na+}^{\prime {}^{\circ}}=n_{Q}\cdot F\cdot \Delta \Phi -n_{Na+}\cdot 2.303\;RT\cdot \Delta pNa\text{.}$$

$n_{Na+}$ is the number of sodium ions transported from one to the other compartment, $n_{Q}$ is the stoichiometric coefficient of the transported charges*,* ΔΦ is the membrane potential difference (here we assume ΔΦ=−150mV) and $\Delta pNa=\log_{10}\left({[Na}_{peri}^{+}]/{[Na}_{cyto}^{+}]\right)$ quantifies the concentration differences of Na^+^ between periplasm and cytosol. Herein we asume ΔpNa=0.5, analogous to typical ΔpH values of 0.5 [57] used when calculating the proton-motive force in other species. For the ATPase ($n_{Na+}=n_{Q}=3.3)$ [58] this results in a value of $\Delta G_{Na+}^{\prime {}^{\circ}}=-9.41$ kJ mol^−1^. Likewise, for the RNF complex ($n_{Na+}=n_{Q}=2$ and multiplication with −1 because the ATPase runs in reverse direction) we obtain $\Delta G_{Na+}^{\prime {}^{\circ}}=5.70$ kJ mol^−1^.

For reactions, where a $\Delta G^{\prime }{}^{\circ}$ could not be determined, we assumed $\Delta G^{\prime }{}^{\circ}=0$ and no thermodynamic constraints were added for pseudo reactions involved in biomass synthesis (see Supplementary Table 1).

A first TFBA simulation of the built stoichiometric *A. woodii* model with integrated $\Delta G^{\prime }{}^{\circ}$ values resulted in an infeasible model. This occurs frequently in reconstructed metabolic models with thermodynamic constraints and is often related to unrealistic $\Delta G^{\prime }{}^{\circ}$ values for some (typically anabolic) reactions. We applied a thermodynamic bottleneck search and correction as described in Ref. [52]. In total, we found 7 bottleneck reactions preventing growth and relaxed their $\Delta G^{\prime }{}^{\circ}$ (see Supplementary Table 1).

#### 2.1.3 Concentration ranges

For the lower and upper concentration bounds of internal metabolites in eq. (4) we used typical values of $10^{-6}$ M and ${2.0\cdot 10}^{-2\;}$M, respectively. The concentrations of dissolved H_2_ and CO_2_ in the medium were set based on calculations presented in the Results section. For each of the three major redox cofactors (Fdx, NAD(H), and NADP(H)), we assumed that the ratio of the reduced and oxidized form lies between 0.01 and 100. For example, for the NAD(H) pool, using the logarithmic concentration values $x_{NADH}$ and $x_{{NAD}^{+}}$, this constraint can be formulated in a linear fashion via:

$$\ln\left(0.01\right)=-4.61\leq x_{NADH}-x_{{NAD}^{+}}\leq 4.61=\ln\left(100\right)$$

### 2.2 Model implementation and simulation

All models were implemented using COBRApy [59] and analyzed with dedicated Python scripts. CPLEX (Version 22.1.1.0) with docplex (Version 2.30.251) was used as solver. In addition, Escher maps [60] were created and used within the CNApy software [61] to visualize (and further analyze) calculated solutions. All models (in SBML and CNApy format), scripts and data used in this work are publicly available via GitHub (https://github.com/klamt-lab/Modeling_Awoodii) and archived on Zenodo (https://doi.org/10.5281/zenodo.18678845).

## 3 Results

### 3.1 Building and analyzing an extended metabolic model of *A. woodii*

#### 3.1.1 Model construction

We aimed to build a well-curated stoichiometric metabolic model of *A. woodii* that covers all pathways of the central metabolism of this bacterium, the anabolic routes to monomers (amino acids, nucleotides, etc.), and the (pseudo) reactions consuming the monomers to synthesize the macromolecules (proteins, RNA, etc.) making up the biomass. As starting point, we used a previously published smaller model of *A. woodii* [45] in the extended version presented in Ref. [26]. Both these models are neither charge- nor mass-balanced, focused on the central metabolism and included only a simplified biomass synthesis reaction taken from an *E. coli* model. The anabolic pathways and the biomass synthesis reaction in the model constructed herein were taken and adapted from a metabolic model of the acetogen *Clostridium autoethanogenum* [40,41]. Specific details and scripts used for the reconstruction of the model can be found in the associated GitHub repository (see Methods).

**Fig. 1:**
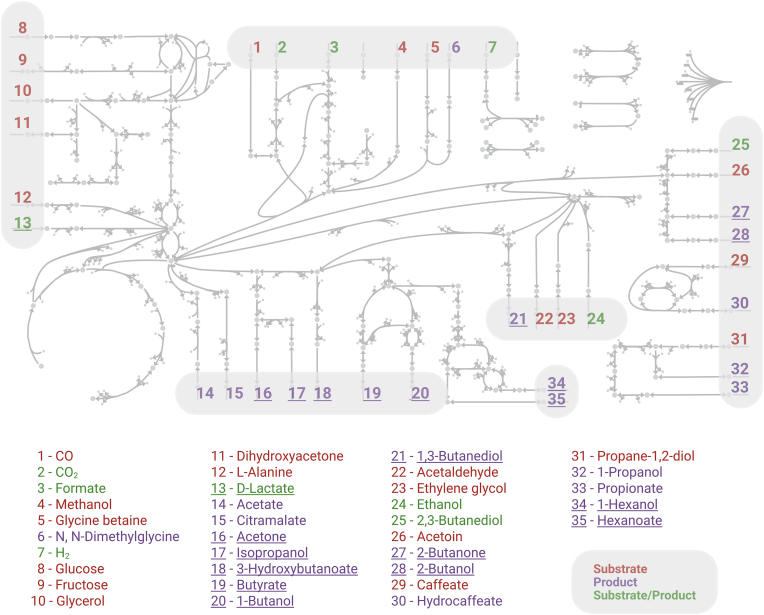
**Substrates and products of the *A. woodii* model (see also**Supplementary Table 2**).** Underlined text indicates a heterologous product.

The resulting model contains 563 reactions and 521 metabolites. The model incorporates exchanges for 13 native substrates and 6 native products. In addition, 6 compounds can serve as substrate or product. The model also includes exchanges and associated heterologous pathways for 11 non-native products. A sketch of the central part of the network with all products and substrates is given in Fig. 1 and a detailed list of substrate and products is provided in Supplementary Table 2.

#### 3.1.2 Initial FBA simulations and validation of the stoichiometric model

For a first simple FBA simulation of the wild-type growth behavior of *A. woodii* on a CO_2_/H_2_ gas mixture we limited the specific uptake rate of H_2_ to 120 mmol g_DW_^−1^h^−1^ (which is in the range of maximal H_2_ uptake rates reported by Novak et al. [21]) and of CO_2_ to 60 mmol g_DW_^−1^h^−1^. All other substrate uptake reactions were closed (rates set to zero). Maximizing the growth rate in this model resulted in $\mu_{\max}=0.107\;h^{-1}$, which is close to measured growth rates of *A. woodii* under these conditions (typically ∼ $0.1\;h^{-1}$ [21,22]).

Afterwards we used data from different chemostat experiments [26] and performed metabolic flux analysis calculations in combination with methods to resolve flux inconsistencies [62]. A summary of these comparisons is shown in Supplementary Table 3 indicating that the model predictions are largely consistent with measured growth as well as substrate uptake and product excretion rates in almost all cases. For example, for growth on CO_2_ and H_2_, only minor changes in the measured CO_2_ uptake and acetate excretion rates were necessary (both less than 3 %, lying within the standard deviations) to obtain a consistent flux distribution with the measured fluxes in the model. Some smaller inconsistency were only detected for experiments with formate as substrate, which is probably caused by carbon imbalances in the data due to the use of yeast extract in the experiments in Ref. [26].

#### 3.1.3 Thermodynamic flux balance analysis

As a next step to validate our metabolic model of *A. woodii* we performed thermodynamic flux balance analysis (TFBA), which, in addition to stoichiometric constraints, takes also thermodynamic constraints into account (Methods). This required the computation and inclusion of the standard Gibbs free energies of reactions ($\Delta_{r}G^{\prime 0}$) in the model together with setting the allowed metabolite concentration ranges (see Methods for details).

##### 3.1.3.1 Concentration bounds for dissolved H_2_ and CO_2_

For TFBA simulation of growth of *A. woodii* under conditions that involve uptake of H_2_ or/and CO_2_, the assumed ranges for dissolved H_2_ and CO_2_ concentrations are crucial. Dissolved gas concentration values in gas fermentation experiments are often reported as partial pressures in the headspace and must therefore be converted to dissolved gas concentrations in water. Generally, the equilibrium concentration $c_{l}^{*}$ of a gas in the liquid phase can be calculated from the partial pressure p in the head space using Henry's law:

(8) $$c_{l}^{*}=p\cdot H^{cp}$$

$H^{cp}$ is the Henry's law constant, which is specific for the respective gas and also dependent on the temperature (here 298.15 K): ${7.8\cdot 10}^{-9}\frac{M}{\text{Pa}}$ for H_2_, ${3.3\cdot 10}^{-7}\frac{M}{\text{Pa}}$ for CO_2_ and ${9.7\cdot 10}^{-9}\frac{M}{\text{Pa}}$ for CO [63]. As a very conservative estimation for the concentration upper bounds, we could assume 100 % partial pressure of the respective gas at standard atmospheric pressure (101,325Pa) and would obtain an equilibrium concentration of ${7.9\cdot 10}^{-4}$ M for H_2_, ${3.3\cdot 10}^{-2}$ M for CO_2_ and ${9.8\cdot 10}^{-4}$ M for CO. However, application of Henry's law requires equilibrium between the gas and the liquid phase, which can generally not be assumed here due to the biomass in the medium consuming the gases. For example, the dynamic change in the dissolved H_2_ concentration, $c_{l,H_{2}}$, can be described by

(9) $$\frac{dc_{l,H_{2}}}{dt}=k_{L}a\cdot \left(c_{l,H_{2}}^{*}-c_{l,H_{2}}\right)-c_{B}\cdot q_{H_{2}}$$

Here, $c_{l,H_{2}}^{*}$ is the equilibrium concentration computed from Henry's law (8), k_L_a is the volumetric mass transfer coefficient, $c_{B}$ is the biomass concentration, and $q_{H_{2}}$ the specific H_2_ consumption rate of the bacteria. In (quasi) steady state we can set $\frac{dc_{l,H_{2}}}{dt}=0$ and solve for the gas concentration:

(10) $$c_{l,H_{2}}=\frac{k_{L}a\cdot c_{l,H_{2}}^{*}-c_{B}\cdot q_{H_{2}}}{k_{L}a}$$

It thus follows that the true dissolved H_2_ concentration is always lower than the equilibrium concentration predicted by Henry's law if there is biomass in the reactor consuming H_2_. In particular, the higher the biomass concentration the larger is that difference. Moreover, when the biomass concentration reaches the critical value $c_{B,crit}=\frac{k_{L}a\cdot c_{l,H_{2}}^{*}}{q_{H_{2}}}$, the dissolved H_2_ concentration becomes zero. For example, a H_2_ partial pressure of 46 Pa was reported by Laura and Jo in Ref. [64] as the minimum required by *A. woodii* for growth. Only if a biomass concentration of (close to) zero is assumed, this value would correspond to a dissolved H_2_ concentration of ${c_{l,H_{2}}^{*}=3.46\cdot 10}^{-7}$ M computed via Henry's law for this partial pressure. In fact, assuming a $k_{L}a$ of 120 h^−1^ (estimated in our lab in a bioreactor with *T. kivui* growing on syngas [65]) and a H_2_ consumption rate of *A. woodii* of 85.6 x $10^{-3}$ mmol g_DW_^−1^ h^−1^ (table 4 in Ref. [21]) we find that the biomass concentration must have been below the extremely small critical value of $c_{B,crit}$ = 0.0005 g_DW_ L^−1^ in this experiment, because otherwise the dissolved H_2_ concentration becomes zero. Would we instead assume a partial pressure of 100 % (101,325Pa) for H_2_ in these calculations, the maximal biomass concentration would still be relatively low at a level of 1.11 g_DW_ L^−1^. These considerations illustrate the potential limitations of gas fermentations due to poor solubility of H_2_ and low gas-liquid mass transfers [[66], [67], [68]] and lead to two important conclusions. First, providing H_2_ partial pressures, under which a gas fermentation experiment has been performed, has only limited information regarding the dissolved H_2_ concentration that is “seen” by the bacteria because it depends, apart from the Henry constant, on many other factors such as biomass concentration, $k_{L}a$ and $q_{H_{2}}$ (which should be provided whenever possible). Second, the dissolved H_2_ concentration is most likely significantly below the value predicted by Henry's law. For example, assuming a biomass concentration of $c_{B}=1$ g_DW_ L^−1^ and then again $k_{L}a=$ 120 h^−1^ and $q_{H_{2}}=85.6$ mmol g_DW_^−1^ h^−1^, the dissolved H_2_ concentration, even for 100 % H_2_ under standard conditions, would be $7.67\cdot 10^{-5}$ M and thus more than one order of magnitude lower than the value predicted by Henry's law. For CO_2_, the effect is less pronounced: using a typical partial pressure of 20 % for CO_2_, a specific CO_2_ uptake rate of 39.7 mmol g_DW_^−1^ h^−1^ (table 4 in Ref. [21]), and the same $k_{L}a$ and biomass concentration as before, we obtain a dissolved CO_2_ concentration of 6.27 x $10^{-3}$ M.

In the calculations presented below, we followed a conservative scenario assuming the case of a biomass concentration close to zero and thus high concentration upper bounds, directly taken from Henry's law. Accordingly, the concentration range for dissolved H_2_ is set to [$10^{-7},{7.9\cdot 10}^{-4}]\;M$ and for CO_2_ to [$10^{-6},{3.3\cdot 10}^{-2}]\text{.}$ However, when discussing results in connection with fermentation processes, the more realistic upper bounds calculated above for a biomass concentration of 1 g_DW_ L^−1^ will be taken into account, leading to a concentration range of [$10^{-7},{7.67\cdot 10}^{-5}]\;M$ for H_2_ and [$10^{-6},6.27\cdot \;10^{-3}]$ M for CO_2_. Generally, higher gas concentrations could be reached in high-pressure gas fermentations. However, we did not consider these conditions because high pressures can lead to reduced biomass synthesis [69] and because many of our analyses aim to shed light on thermodynamic bottlenecks in the wild type under standard conditions.

##### 3.1.3.2 Thermodynamic FBA and MDF calculations

**Fig. 2:**
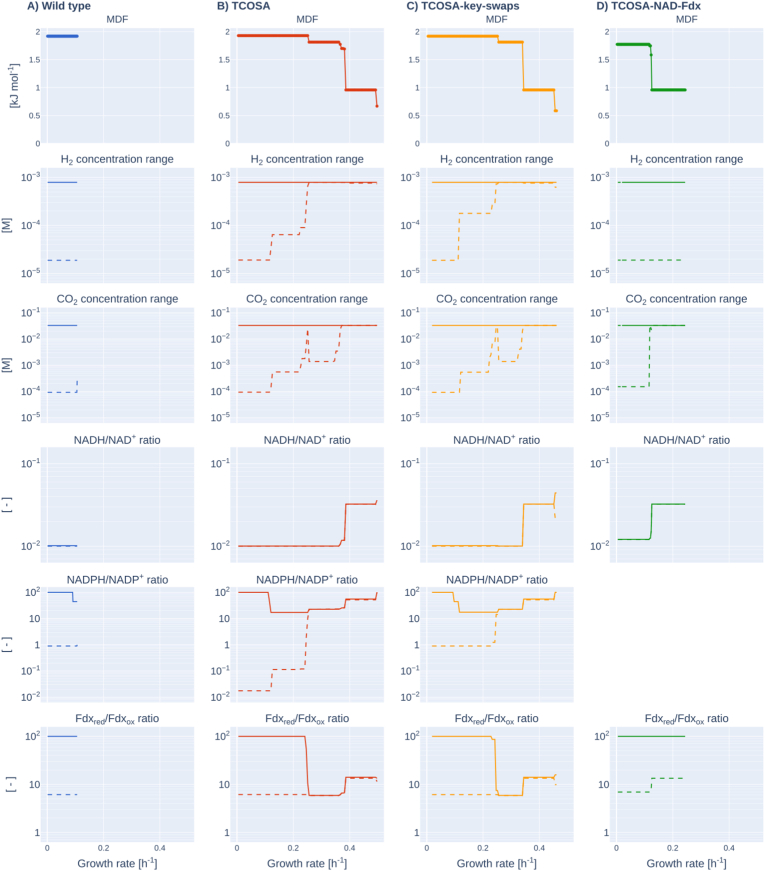
**Results from MDF maximization for increasing growth rates (stepsize: 1 % of maximal growth rate) in the four different model variants considered: A) wild type, B) TCOSA, C) TCOSA-key-swaps, and D) TCOSA-NAD-Fdx.** The results were computed as follows: for a given model and (fixed) growth rate, the MDF was maximized (results shown in the first row). Afterwards, the found optimal MDF was additionally fixed and the minimal value (dashed line) and maximal value (solid line) for the H_2_ and CO_2_ concentrations and for the three cofactor ratios were determined before proceeding with the next growth rate.

**Fig. 3:**
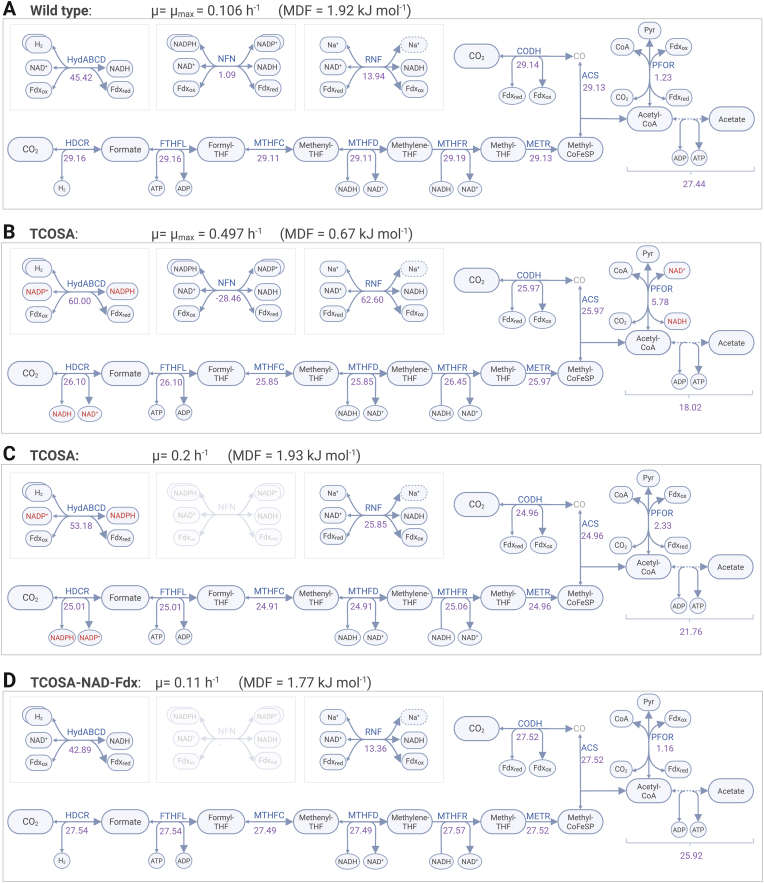
**Thermodynamic driving forces, fluxes of key reactions and redox cofactor swaps for four selected scenarios from**Fig. 2**.** The results were obtained by maximizing the MDF for the given growth rate and model variant (the obtained MDF is shown on top of each box). For the TCOSA solutions (B)-(D), an additional optimization was performed in a second step to find the minimal number of swaps needed to reach the respective maximal MDF. Red cofactor names indicate cofactor swaps. Purple numbers are the fluxes of the respective reactions. See also Fig. 2.

Based on the settings described above, we performed a TFBA maximizing the growth rate and found that the resulting $\mu_{\max}=0.107\;h^{-1}$ coincides with the result obtained with classical FBA, thus indicating thermodynamic feasibility of this flux distribution. In order to assess the maximal thermodynamic potential associated with the growth-optimal flux distribution, we determined its max-min driving force (MDF; see Methods). Since we demanded in the TFBA calculation a minimum driving force of $f_{i,\min}=0.1$ kJ mol^−1^ for all active reactions i (eq. (6)), the MDF cannot fall below this value. Indeed, Fig. 2A shows that the MDF at the maximal growth rate is 1.92 kJ mol^−1^, which indicates a sufficient thermodynamic span for all active reactions and flexibility in the metabolite concentrations to realize the associated flux distribution (key fluxes are shown in Fig. 3A; the entire set of fluxes can be found in Supplementary Table 4). We then computed the MDF also for growth rates below $\mu_{\max}$, which would allow the usage of alternative pathways with lower biomass yields but potentially increased MDF values. However, we found for all growth rates the same MDFs (Fig. 2A), i.e., pathways with reduced biomass yields cannot enhance the MDF.

Next, we computed feasible ranges for the oxidation states of the three major redox cofactors in the growth-optimal solution with maximal MDF and found that the NADH/NAD ^+^ ratio must be *below* 0.0103, hence, the NAD(H) cofactor pool must be almost entirely in the oxidized form NAD^+^ (>98 %). In contrast, the NADPH/NAPD ^+^ ratio must lie *above* 0.877 (>46 % reduced) and that of Fdx_red_/Fdx_ox_ even above 6.089 (>85 % reduced) to enable high growth rates with maximal driving force. In particular, the highly reduced state of ferredoxin is required to ensure thermodynamic feasibility of the CODH and, together with the more than 590-fold more oxidized state of the NAD(H) pool, a high driving force for the RNF complex. A sufficiently reduced NADP(H) pool ensures that anabolic reactions using NADPH as electron donor can run with high driving forces.

##### 3.1.3.3 Influence of H_2_ and CO_2_ concentrations on maximal growth rate

The highest feasible growth rate (and its associated MDF value) determined in the TFBA calculation depend on the chosen upper bounds for the dissolved H_2_ and CO_2_ concentrations. Using a concentration variability analysis (see Ref. [53]), we determined the minimal and maximal dissolved H_2_ and CO_2_ concentrations under which the respective growth rate and its (maximal) MDF could be reached (Fig. 2A). Apparently, relatively large concentration ranges are possible with minimal concentrations of ∼10^−4^ M for CO_2_ and ∼10^−5^ M for H_2_, which indicates robust behavior against concentration fluctuations.

**Fig. 4:**
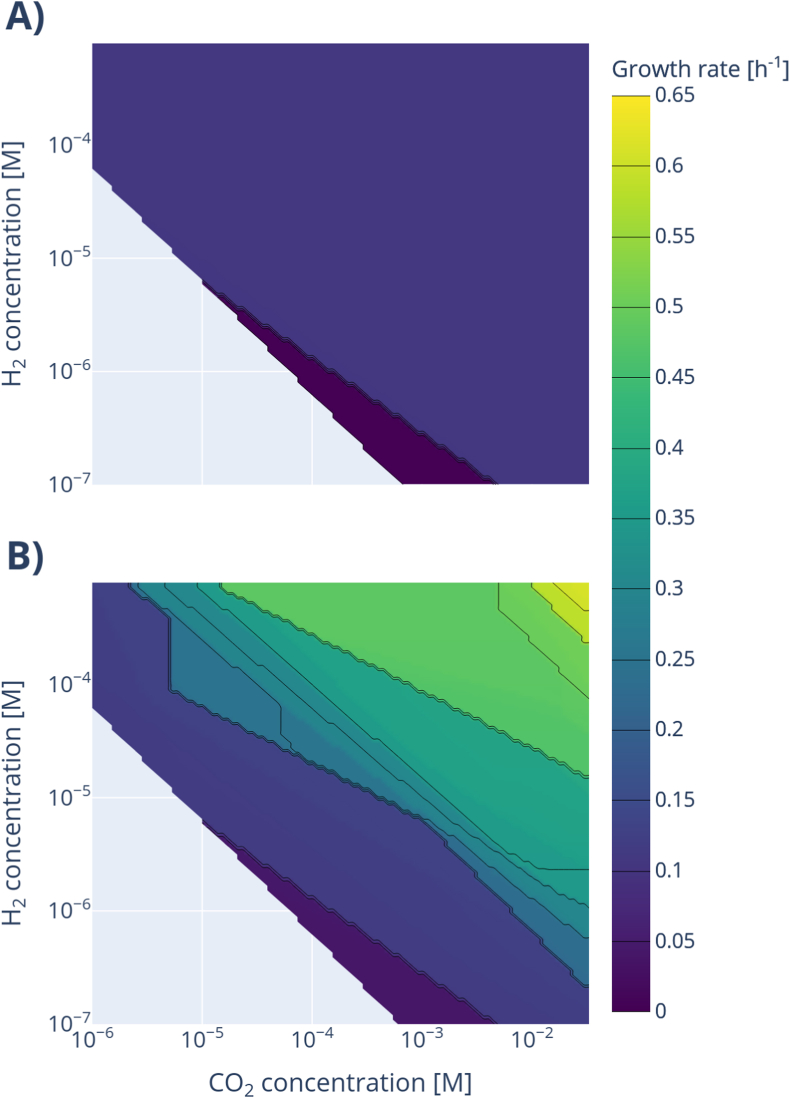
**Maximal growth rates achievable under different dissolved H_2_ and CO_2_ concentrations in the medium in the wild-type model (A) and in the TCOSA model (B).** In each simulation, a minimal MDF of 0.1 kJ mol^−1^ was demanded. The grey regions indicate concentration ranges, where neither growth nor net ATP synthesis is feasible. Note that the predicted feasible concentration ranges are larger than in Fig. 2A, because here only a minimal MDF of 0.1 kJ mol^−1^ was demanded while in Fig. 2A the maximal MDF for the respective growth rate was considered.

For a more thorough analysis, we next discretized the original H_2_ and CO_2_ concentration ranges in equidistant steps and computed for each combination the resulting $\mu_{\max}$ via TFBA, thereby demanding a minimal MDF of 0.1 kJ mol^−1^. Thus, instead of maximizing the MDF for a given growth rate used in the calculations above, we here computed the maximal growth rate feasible for a given H_2_/CO_2_ concentration under some minimal driving force. The predicted minimal concentration values should therefore be seen as rather optimistic lower bounds (and the critical concentrations are likely higher in reality). The results are displayed in the two-dimensional contour plot in Fig. 4A showing two major isoclines along which growth becomes (in)feasible or where a switch to another metabolic mode with lower/higher growth rate occurs, respectively. It can clearly be seen that, at the boundary of the feasible concentration ranges, reducing the concentration of one gas must be counteracted with a higher concentration of the other to maintain growth. It is again apparent, that the maximal growth rate ($\mu_{\max}=0.107\;h^{-1}$) can be reached over a broad range of H_2_ and CO_2_ concentrations indicating a robust operation of the metabolism also under fluctuating gas concentrations.

### 3.2 Systematic analysis of the effects of cofactor swaps

#### 3.2.1 Construction of a TCOSA model for *A. woodii*

**Fig. 5:**
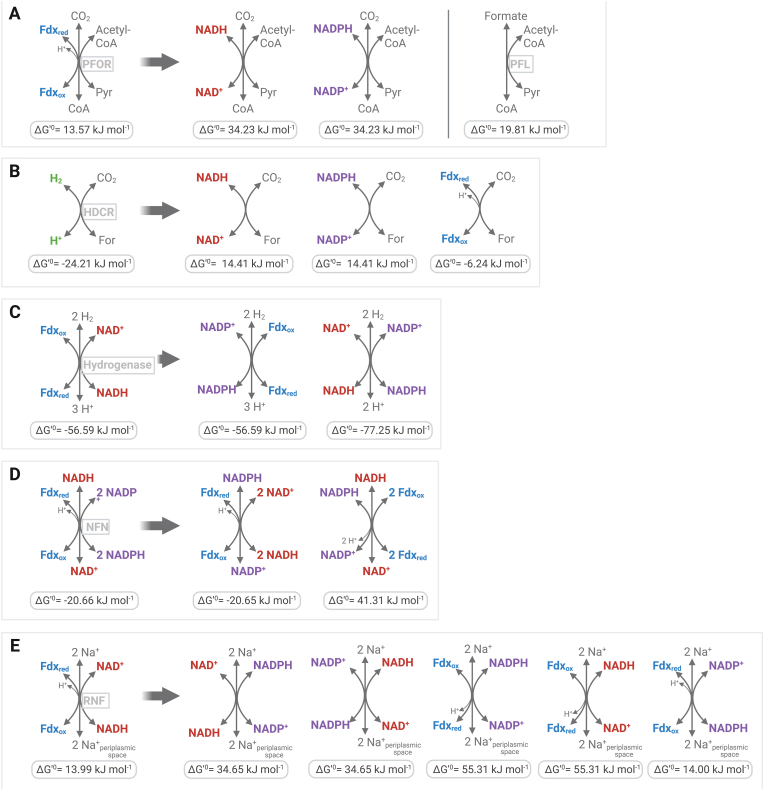
**Different redox cofactor variants (cofactor swaps) for five selected redox reactions in *A. woodii*.** On the left-hand side, the original cofactor specificity of the respective enzyme in *A. woodii* is shown. The variants on the other side of the arrow are obtained by cofactor swaps and recalculation of the $\Delta G^{'}{}^{\circ}$.

In the next step, we aimed to use the model to analyze the optimality of the wild-type redox cofactor specificities and the effect of potential redox cofactor swaps on thermodynamic driving forces and biomass yields. For this purpose, we utilized the recently introduced TCOSA (Thermodynamics-based COfactor Swapping Analysis) approach [52]. Given a constraint-based metabolic model, the TCOSA method starts with a preprocessing step: each redox reaction involving a redox cofactor (NAD(H) or NADP(H) in the *E. coli* model investigated in Ref. [52]) is duplicated in the model and the cofactor swapped in the duplicated reaction. Herein we had to deal with the more complicated case of three major redox cofactor pools, namely NAD(H), NADP(H) and Fdx. Hence, for a redox reaction using one of the three redox cofactors, two copies (one for each of the other two cofactors) have to be added to the *A. woodii* TCOSA model to obtain all three variants. This is exemplified in Fig. 5A for the PFOR reaction. In this particular case, in addition to the two standard cofactor swaps, we also included the pyruvate formate lyase (PFL) reaction in the TCOSA model (Fig. 5A), which could take over the role of the PFOR reaction. There is indeed evidence that the gene for this enzmye is present in *A. woodii* (though it is expressed only in small amounts with H_2_ + CO_2_ as substrate [26]) and that heterologous expression of *pfl* from *A. woodii* can enhance growth of another acetogen, *Clostridium ljungdahlii*, on formate [70].

There are several other cases that need special treatment. For the hydrogen-dependent CO_2_ reductase (HDCR) reaction, it makes sense to consider a potential replacement of the electron donor H_2_ with one of the reduced forms of the three redox carierers (Fig. 5B) giving rise to a total of four variants for the HDCR reaction. In the variants where NADPH or NADH is used instead of H_2_, the HDCR becomes effectively a formate dehydrogenase. In the case of the hydrogenase reaction (2H_2_ + NAD^+^ + Fdx_ox_ → 3 H^+^ + NADH + Fdx_red_), we do not consider the replacement of H_2_, however, since two redox carriers (NAD^+^ and Fdx) are involved, in total 3 variants can be built (Fig. 5C). Another special case is the bifurcating NFN reaction, in which all three redox coafactors are involved (Fdx_red_ + H^+^ + NADH + 2 NADP^+^ → Fdx_ox_ + NAD^+^ + 2 NADPH), again giving rise to two additional variants (Fig. 5D). Finally, for the Na^+^-pumping RNF complex with two involved redox cofactors one can even construct five additional combinations yielding six variants in total (Fig. 5E). Importantly, for all reaction variants constructed, the standard Gibbs free energy change ($\Delta G^{'}{}^{\circ}$) was recalculated (Fig. 5). In total, 146 variants of 72 reactions involving redox cofactors (excluding heterologous reactions) were added in the *A. woodii* TCOSA model. As in the original TCOSA framework, for a given redox reaction, the model was constraint to use at most one variant of that reaction in a calculated flux distribution.

#### 3.2.2 What would be the optimal redox cofactor specificity in *A. woodii* for growth?

With the constructed TCOSA model of *A. woodii*, we can now determine (a) the maximal thermodynamic potential (MDF) in the network for a given growth rate, and (b) the maximal thermodynamically feasible growth rate when allowing changes in the redox cofactor specificities and then compare these values with the wild-type model. To address these questions, we scanned again the MDF for increasing growth rates in the full TCOSA model (Fig. 2B), similar as we did for the wild-type model (Fig. 2A).

A first observation is that the maximal growth rate could, in principle, be increased up to 0.497 h^−1^, although with a significant reduction in the achievable MDF (0.67 kJ mol^−1^). This growth-optimal TCOSA model solution requires 13 cofactor swaps, of which the following three can be identified as key swaps (see Fig. 3B and Supplementary Table 4):

(i) The HDCR reaction uses NADH instead of H_2_ (variant 1 in Fig. 5B), hence, it then represents a formate dehydrogenase.
(ii) The bifurcating hydrogenase reaction reduces Fdx_ox_ and NADP ^+^ instead of Fdx_ox_ and NAD^+^ (variant 1 in Fig. 5C).
(iii) The PFOR reaction uses NADH instead of Fdx (variant 1 in Fig. 5A).

These three cofactor swaps, of which (i) and (iii) are rather hypothetical variants, whereas (ii) corresponds to a natively occurring reaction (e.g., in *C. autoethanogenum*), have one common goal, namely to provide the largest possible amount of reduced Fdx for ATP synthesis via the Na^+^-dependent RNF and ATPase complexes (Fig. 3B). Through the first cofactor swap, H_2_ is saved in the HDCR reaction, which can then exclusively be used in the modified bifurcating hydrogenase reaction to reduce more ferredoxin and NADPH. In addition, a portion of the NADPH now produced in the modified bifurcating hydrogenase reaction can be used to drive the bifurcating NFN reaction in reverse direction (Fig. 3B) providing additional reduced ferredoxin (plus NADH). Lastly, using NADH instead of Fdx_red_ in the PFOR reaction further saves reduced ferredoxin which becomes available for the RNF reaction and thus ATP production increasing the overall biomass yield. The other cofactor swaps in this optimal solution occur in reactions with smaller fluxes and further optimize the ferredoxin and NADP(H)/NAD(H) stoichiometries. For example, one cofactor swap is suggested for the prephenate dehydrogenase (PPND) reaction with the original stoichiometry

prephenate + NAD^+^ → 4-hydroxyphenylpyruvate + CO_2_ + NADH,

which is part of the shikimate pathway. This reaction has a relatively low $\Delta G^{\prime 0}$ of −10.78 kJ mol^−1^ and our thermodynamic analysis suggests that Fdx could be reduced in this reaction instead of NAD^+^, further increasing the amount of Fdx_red_ available for ATP synthesis. However, the flux through this reaction is relatively low (0.21 mmol g_DW_^−1^ h^−1^) and the stoichiometric gain of Fdx_red_ is thus small.

For the growth rates between 0.387 h^−1^ and 0.492 h^−1^, a plateau of larger MDF values (0.958 kJ mol^−1^) can be reached compared to the solution with maximum growth rate (Fig. 2B). In these solutions, only the first two of the three key swaps listed above are used (no swap in the PFOR reaction). Generally, while our analysis indicates that operation of the described metabolic modes with a four-to fivefoled increased growth rates compared to the WT (μ > 0.387 h^−1^) would, in principle, be feasible, their operation requires (a) an extremely reduced NADP(H) pool (NADPH/NADP^+^ > 50) in order to drive the NFN reaction in reverse direction, and (b) very high concentrations of dissolved CO_2_ and H_2_ to drive the altered HDCR, bifurcating hydrogenase, and PFOR reactions. In fact, the required CO_2_ and H_2_ concentrations approach the upper bounds used in the model (see Fig. 2B and 4B). This indicates that a stable operation of those metabolic modes, especially under fluctuating environments and with higher biomass concentrations (where the CO_2_ and H_2_ concentrations are lower than the used upper bounds; see above), is likely not feasible. For similar reasons, solutions on the next higher MDF plateau (growth rates between 0.26 h^−1^ and 0.36 h^−1^; MDF∼1.813 kJ mol^−1^) are unlikely to be robust enough.

The situation is somewhat different for growth rates between 0.11 h^−1^ and 0.25 h^−1^, which are all higher than for the wild-type and even allow slightly higher MDF values (1.93 kJ mol^−1^ vs. 1.92 kJ mol^−1^ in the wild-type model). An exemplary solution is depicted in Fig. 3C for the growth rate of 0.20 h^−1^ (the complete flux distribution is given in Supplementary Table 4). From the three major cofactor swaps in the TCOSA growth-optimal solution discussed above, the swap in the bifurcating hydrogenase reaction is maintained, the HDCR reaction is again replaced with a formate dehydrogense, but now using NADPH instead of NADH (this variant is natively used by the actogens *M. thermoacetica* [1]), and PFOR is again not swapped. While these solutions are feasible with lower CO_2_ and H_2_ concentrations compared to the TCOSA solutions with the highest growth rates, they are still 0.5 orders of magnitude higher than for the growth rates achievable with the wild type (Fig. 2B). It can only be speculated whether such a solution could enable a robust operation under a wider range of relevant conditions, including those prevailing in a bioreactor.

Finally, an important observation can be made in the TCOSA model when focusing on growth rates that are also possible with the wild-type model (0 < μ < 0.107 h^−1^): in those cases, the MDF in the TCOSA model can be increased only to a very minor extent compared to the wild-type model (from 1.92 to 1.93 kJ mol^−1^; Fig. 2B). This indicates that the evolved redox cofactor specificites in *A. woodii* are close to being optimal in terms of the achievable network-wide thermodynamic driving force for these growth rates.

Similar as discussed for the wild-type, Fig. 4B depicts the maximally achievable growth rates with the redox cofactor swaps in the TCOSA model in dependence of the CO_2_ and H_2_ concentrations. Again, in these computations, only a small minimum network-wide driving force (MDF) of 0.1 kJ mol^−1^ was demanded. i.e. the computed values represent rather optimistic upper bounds for the growth rates. It can be seen that TCOSA solutions with higher growth rates would require quickly increasing CO_2_ and H_2_ concentrations, which become then unrealistic for natural environments. It should also be noted that the isoclines in Fig. 4B often separate regions with different cofactor swaps. Hence, switching between these solutions would require changing cofactor specificities, which is either not possible or would require to invest in alternative enzyme variants and then switching their expression.

#### 3.2.3 A TCOSA model allowing only key cofactor swaps

As was mentioned above, in the solutions with higher growth rates in the TCOSA model, we found that only few (key) redox cofactor swaps in central redox reactions appear essential, while many other suggested swaps occur in reactions with small fluxes and are thus likely to have only minor effects. To test the hypothesis, we considered a reduced TCOSA model where redox cofactor swaps were only allowed in the five reactions listed in Table 1 representing key swaps found in many solutions in the TCOSA model. Four of these reactions are located in the WLP, while the fifth is the PFOR reaction, which can be exchanged by a PFL reaction. For several of these cofactor swaps, enzymes catalyzing the reaction variant with the altered cofactor(s) are either known from other acetogenic species (e.g. replacing the HDCR by an NADPH- or NADH-dependent formate dehydrogenase or an NADP^+^-reducing hydrogenase; see Discussion) or an engineering (or evolution) of enzymes using the alternative cofactor appears possible. We call this model, which we will later also use for screening production capabilities of *A. woodii* mutants, TCOSA-key-swaps. In this model, we reperformed the MDF scan for increasing growth rates as done before for the wild-type and full TCOSA model revealing that the limited set of selected redox cofactor swaps shows simlar characteristics as the full TCOSA model (Fig. 2C). The maximally achievable growth rate and the MDF values associated with the different growth rates are only slighltly reduced. Thus, the cofactor swaps included in the TCOSA-key-swaps model are sufficient to generate a solution space that covers the most relevant part of the TCOSA model.

**Table 1:** Overview of allowed cofactor swaps in the TCOSA-key-swaps model.

Reaction ID	Reaction name	Native cofactor	Alternative reaction ID	Alternative cofactors
HDCR	Hydrogen-dependent CO_2_ reductase	H_2_	HDCR_VARIANT_FDX HDCR_VARIANT_NADH (formate dehydrogenase) HDCR_VARIANT_NADPH (formate dehydrogenase)	Fdx NADH NADPH
HydABCD	Electron-bifurcating hydrogenase	Fdx + NADH	HydABCD_VARIANT_NADPH	Fdx + NADPH
MTHFDnad	Methylene-THF dehydrogenase	NADH	MTHFDnad_VARIANT_NADPH	NADPH
MTHFR2	Methylene-THF reductase	NADH	MTHFR2_VARIANT_NADPH	NADPH
PFOR	Pyruvate-ferredoxin oxidoreductase	Fdx (+CO_2_)	PFL (Pyruvate formate lyase)	Formate

#### 3.2.4 Does *A. woodii* need three cofactor pools for growth?

Next, we used the full TCOSA model to investigate whether the three redox cofactors pools (NAD(H), NADP(H) and Fdx) in the central metabolism of *A. woodii* are mandatory for growth or whether, at least theoretically, two redox cofactors could enable growth fulfilling all thermodynamic constraints. In principle, three cases with two redox cofactor pools can be considered: NAD(H) and NADP(H) (denoted with TCOSA-NAD-NADP), NAD(H) and Fdx (denoted with TCOSA-NAD-Fdx), and NADP(H) and Fdx (denoted with TCOSA-NADP-Fdx). We excluded the case TCOSA-NADP-FDX because the redox couples NAD^+^/NADH and NADP^+^/NADPH have the same redox potential, hence, identical results would be obtained for TCOSA-NAD-FDX and TCOSA-NADP-FDX.

We first analyzed the case TCOSA-NAD-NADP. We disabled all reactions in the (full) TCOSA model that use ferredoxin and maximized again the MDF value for increasing growth rates. Even for small growth rates no solutions could be found that are thermodynamically feasible (MDF ≥ 0.1 kJ mol^−1^). Hence, the use of ferredoxin as a redox cofactor with low standard redox potential is mandatory.

We therefore focused on the case TCOSA-NAD-Fdx (Fig. 2D). We disabled all NADP(H)-dependent reactions in the TCOSA model and determined, for increasing growth rates, MDF-maximal flux distributions, where NADP(H)-dependent reactions in the wild type are replaced with NAD(H)- or Fdx-dependent variants (plus possibly further cofactor swaps between NAD(H) and Fdx). Unexpectedly, we found that there are not only solutions with growth but even solutions with higher growth rates than for the wild type ($\mu_{\max}=0.244\;h^{-1}$, Fig. 2D). The solutions with higher growth rates (μ > $0.12\;h^{-1}$) reach smaller MDF values of 0.958 kJ mol^−1^ and swap again the HDCR reaction with a NADH-dependent formate dehydrogenase. As in the TCOSA model, this solution requires very high levels of CO_2_ and may thus not be robust enough for realistic environmental conditions. More relevant are therefore solutions with growth rates that are also achievable with the wild-type model. Here, larger MDF values close to the one of the wild type (1.77 vs. 1.92 kJ mol^−1^) can be reached and wider concentration ranges are possible (Fig. 3D depicts a representative solution for μ = $0.11\;h^{-1}$; see Supplementary Table 4 for the complete set of fluxes). Interestingly, none of the key swaps found in previous calculations is used in this solution; all 23 reactions with cofactor swaps carry only small fluxes (all below 0.8 mmol g_DW_^−1^ h^−1^). They mainly replace NADP(H) with either NAD(H) or Fdx in such a way that a thermodynamically feasible flux distribution can be generated. The NFN reaction (requiring all three redox cofactors) cannot run and carries thus a zero flux. This bifurcating reaction, which is normally used by *A. woodii* to adjust the stoichiometric ratio of produced NADH and NADPH, is simply not needed anymore when there is no NADP(H) pool. This comes with the advantage that no Fdx_red_ is consumed in the NFN reaction. A smaller percentage of the saved Fdx_red_ molecules is now consumed in few redox reactions, which formerly used NADPH and require an electron donor with low redox potential. The remaining Fdx_red_ can be used for ATP synthesis via the RNF and ATPase complexes. This operation even enables a slightly higher biomass yield and growth rate compared to the wild type (up to μ $∼\;0.12\;h^{-1}$) with an only minor reduction in the MDF. One may again speculate why such a solution has not been favored by nature. One possible reason is that 23 reactions would require a cofactor swap (the majority would replace NADP(H) with NAD(H) and only a few with Fdx), mostly in biosynthetic pathways that have evolved over millions of years. Furthermore, while the found solution with Fdx and NAD(H) as redox cofactor pools might work for chemolithoautotrophic conditions, H_2_ is not available under heterotrophic conditions (e.g., with sugars as substrate). A reduction of Fdx would then only be possible via the PFOR reaction (operating in direction of acetyl-CoA), which might not be sufficient to feed all reactions consuming Fdx_red_. As we had previously shown in *E. coli* [52], two redox cofactor pools will be required to enable efficient simultaneous operation of oxidative redox reactions (e.g., in glycolysis) and reduction steps in the anabolism during heterotrophic growth. Hence, the classical partition into the oxidized NAD(H) and reduced NADP(H) pool would then be needed. It might therefore be the best choice to operate with three distinct redox cofactor pools to establish a flexible and robust redox metabolism under a diverse range of environmental conditions.

### 3.3 Screening the capabilities of *A. woodii* to synthesize chemicals from C1 substrates with and without cofactor swaps

One central goal of this work is systematically analyzing the capability of *A. woodii* to synthesize relevant products from C1 carbon substrates. The recent review of Pettinato et al. [17] already presented results in this direction, but focused on stoichiometric and bioenergetic aspects of bioproduction scenarios. Herein, we extend this analysis by explicitly taking into account thermodynamic constraints (and feasibility), both with and without allowing redox cofactor swaps. Regarding the swaps, in order to avoid too many or unrealistic cofactor changes, we focused on the subset of redox cofactor swaps listed in Table 1, whose implementation appears possible, either by using suitable enzymes from other species or by enzyme engineering. Accordingly, we used the TCOSA-key-swaps model for analyzing the bioproduction potential of *A. woodii*. From the total of 19 substrates included in the *A. woodii* model, we focused on four classical C1 substrates or substrate combinations, respectively, which are most relevant for potential industrial usage: H_2_ + CO_2_, formate, methanol + CO_2_, and methanol + formate. Since we were investigating potential fermentation processes (with significant biomass in the reactor), we used the tighter bounds for the maximal H_2_ and CO_2_ concentrations in the medium, when these compounds served as substrates (see also section 3.1.1). Regarding the maximal substrate uptake rates we assumed for H_2_ + CO_2_ the same bounds as before (120 mmol g_DW_^−1^ h^−1^ for H_2_ and 60 mmol g_DW_^−1^ h^−1^ CO_2_) and for the other three scenarios a maximal total molar carbon uptake of 100 mmol g_DW_^−1^ h^−1^.

From the 22 products in the model (11 native, 11 heterologous), we selected 15 target compounds (4 native, 11 heterologous) considered to be of high potential: 1,3-butanediol, 2,3-butanediol (2,3-BDO), 2-butanol, 3-hydroxybutanoate, acetate, acetone, butanone, butyrate, citramalate, ethanol, hexanoate, hexanol, isopropanol, lactate, and 1-butanol. For four products (1-butanol, 1,3-butanediol, ethanol and hexanol) we considered pathway variants (AOR = aldehyde:ferredoxin oxidoreductase, ALD = aldehyde dehydrogenase, DRA = 2-deoxyribose-5-phosphate aldolase), which were taken from Pettinato et al. [17].

**Fig. 6:**
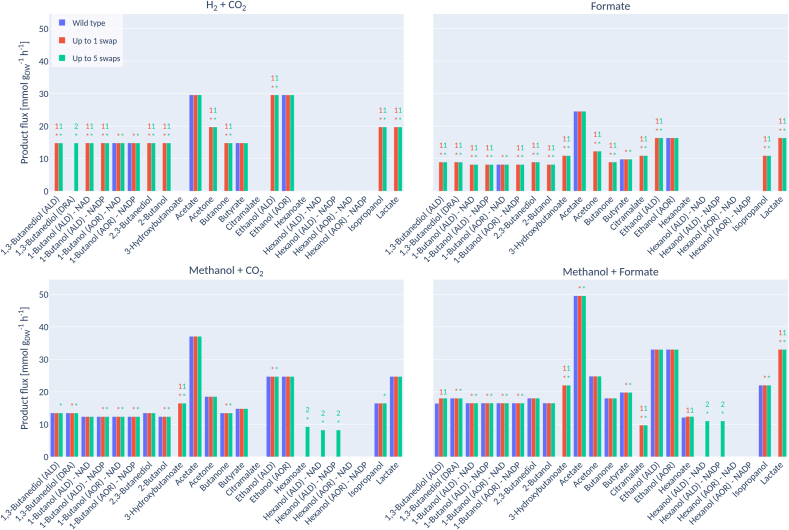
**Maximal production capabilities of *A. woodii* for different substrate-product combinations with and without cofactor swaps.** Four different substrates (or substrate mixtures) were considered: H_2_ (assumed max. uptake rate: 120 mmol g_DW_^−1^ h^−1^) + CO_2_ (max. uptake rate: 60 mmol g_DW_^−1^ h^−1^); formate (max. uptake rate: 100 mmol g_DW_^−1^ h^−1^); methanol (max. uptake rate: 50 mmol g_DW_^−1^ h^−1^) + CO_2_ (max. uptake rate: 50 mmol g_DW_^−1^ h^−1^); methanol (max. uptake rate: 50 mmol g_DW_^−1^ h^−1^) + formate (max. uptake rate: 50 mmol g_DW_^−1^ h^−1^). Three different model variants were used: (1) wild-type model (blue), (2) TCOSA-key-swaps model with one allowed cofactor swap (red), and (3) TCOSA-key-swaps model with up to 5 cofactor swaps (green). In each optimization, we optimized the maximal product synthesis rates under the constraints MDF ≥ 0.1 kJ mol^−1^ and μ ≥ 0.02 h^−1^ with blocked acetate excretion. The red/green number indicates the number of swaps used (if no number is given, no redox cofactor swap could improve the product flux). A red/green asterisk on top of the bar indicates whether the MDF could be improved with swaps (which may involve further swaps compared to the maximization of the product flux). For a detailed list of all solutions see Supplementary Table 5.

For each of the 4 ×23 = 92 substrate-product combinations, the calculations were performed with three different model setups (indicated with blue, red and green color in Fig. 6): (1) wild-type model, (2) TCOSA-key-swaps model with one allowed cofactor swap, and (3) TCOSA-key-swaps model with up to 5 cofactor swaps (one swap allowed for each reaction in Table 1). For each substrate-product combination under the respective model setup, we optimized the maximal product synthesis rates under the constraints MDF ≥ 0.1 kJ mol^−1^ and μ ≥ 0.02 h^−1^. Excretion of acetate was blocked in these calculations, hence, if a solution is found for a particular scenario then it will imply full coupling of growth with production of the respective target chemical. Finally, to assess the thermodynamic driving force we also computed the maximally achievable MDF for the maximal product flux in each scenario (in those cases, further swaps are allowed in the TCOSA models (up to the maximal number of swaps) to reach the highest MDF).

Detailed results of the optimizations performed for all considered scenarios can be found in Supplementary Table 5 and a summary is depicted in Fig. 6. A first observation is that introduction of cofactor swaps increases the number of feasible growth-coupled product syntheses. This is particularly true with formate and H_2_ + CO_2_ as substrates, where the number of feasible cases increases from 4 to 17 (formate) and from 5 to 16 (H_2_ + CO_2_), respectively. For the two scenarios with methanol as (co-)substrate, a larger percentage of the products could already be synthesized without introducing cofactor swaps. For some substrate/product combinations, no feasible solution could be found, even when allowing up to 5 swaps. For example, hexanoate and hexanol cannot be synthesized with formate, neither with nor without swaps. Notably, these two particular cases differ from the results obtained in a purely stoichiometric model [17] and are thus a consequence of taking thermodynamic constraints (demanding MDF ≥ 0.1 kJ mol^−1^) into account.

Generally, in cases where cofactor swaps enable a (otherwise infeasible) growth-coupled synthesis of a target product, a single swap is often sufficient. Exceptions are, for example, the two cofactor swaps needed for enabling hexanol production when using methanol as (co-)substrate. We also found that, in cases where already the wild type is able to synthesize a certain product, cofactor swaps may often enhance the thermodynamic driving force (MDF). For example, with methanol + formate as substrate mixture, isopropanol synthesis is already feasible in the wild type but a cofactor swap in the reaction of the bifurcating hydrogenase (variant 1 in Fig. 5C) increases the MDF from 0.95 to 2.29 kJ mol^−1^ (see Supplementary Table 5). On the other hand, in a few cases, a higher product synthesis achieved through a cofactor swap may be accompanied with a reduced MDF. For example, introducing a swap in the reaction of the bifurcating hydrogenase increases the maximal production flux for 1,3-butanediol (ALD) from 16.41 (wild type) to 18.02 mmol g_DW_^−1^ h^−1^ when using methanol + formate as substrate, however, the MDF reduces then from 0.95 to 0.82 kJ mol^−1^. Reduced thermodynamic driving forces may potentially affect productivity, thus indicating potential trade-offs between product yield and productivity.

For all five reactions listed in Table 1, at least one cofactor swap has been used in at least one particular solution. The two most frequent swaps are (i) the bifurcating hydrogenase variant replacing NAD^+^ with NADP^+^, and (ii) replacing the HDCR by an Fdx-dependent formate dehydrogenase (this swap is especially relevant if formate is used as a (co-)substrate). Hence, depending on the chosen substrates, these variants should have the highest priority when constructing new *A. woodii* (platform) strains with altered redox reactions.

## 4 Discussion

In this study, we presented a large-scale constraint-based metabolic model of *A. woodii* with integrated thermodynamic parameters. This model allowed us to perform a wide range of simulations to investigate (a) the physiology of the redox metabolism as well as (b) the production capabilities of this acetogenic model organism, with a particular focus on thermodynamic driving forces and the effects of potential redox cofactor swaps.

Given the relevance of *A. woodii* as a model organism for acetogens, it appears surprising that only smaller and simplified stoichiometric models of the core metabolism of *A. woodii* have been published so far. Moreover, no *A. woodii* model exists to date that includes thermodynamic parameters and constraints to perform TFBA studies or the calculation of network-wide driving forces (such as the MDF) as used herein. We believe that the explicit consideration of thermodynamic driving forces in metabolic flux calculations is essential for acetogenic organisms, because these species live at the edge of thermodynamic feasibility. Accounting for thermodynamics is also particularly relevant when analyzing their production capabilities to avoid overly optimistic results. While several metabolic (including genome-scale) models have been published for other acetogenic model organisms, thermodynamic parameters and constraints have only rarely been integrated in such models (three exceptions are [67,71,72]. Our study is arguably the most comprehensive systematic investigation of thermodynamic driving forces in acetogenic bacteria in the context of autotrophic growth and potential production scenarios, both with and without inclusion of potential redox cofactor swaps.

Initial (T)FBA simulations with our reconstructed model showed that predicted growth rates and exchange fluxes for growth on different substrates are largely consistent with previously reported data from growth experiments with *A. woodii*. TFBA simulations furthermore revealed that growth-related flux distributions with H_2_+CO_2_ as substrate can reach an MDF of up to 1.92 kJ mol^−1^. These (and other) computed results depend on the dissolved gas concentrations. In this context, Fig. 4 provides a useful resource with upper bounds of possible growth rates for different dissolved H_2_/CO_2_ concentrations. Generally, since the latter depend not only on the partial pressures in the gas phase, but also on the k_L_a and the biomass concentration in the medium, we suggest to provide these values, at least the biomass concentration, when reporting about cultivations of acetogenic species in the lab.

Employing the TCOSA approach in combination with our model, we then investigated the effects of modifying the redox cofactor specificities of redox reactions in the metabolism of *A. woodii*. For growth on H_2_ + CO_2_, the model predicted that appropriate swaps between the three major redox cofactors could enable an up to fivefold increase of the growth rate, but only with very high H_2_ + CO_2_ concentrations that are unrealistic for typical environmental conditions. Also, the associated metabolic flux distributions could operate only within rather small ranges of redox states for the respective cofactors. More interesting are therefore solutions with growth rates that are only twice as high as for the wild-type but reach similar MDF values and are feasible in a much wider range of gas concentrations and cofactor redox states. These solutions involve two key cofactor swaps: NADPH replaces H_2_ in the HDCR reaction (which then becomes a formate dehydrogenase (Fdh) reaction) and NADP^+^ replaces NAD^+^ as electron acceptor in the bifurcating hydrogenase reaction. In fact, corresponding enzymes with these cofactor specificities are known from other acetogens, e.g. the NADPH-dependent Fdh in *M. thermoacetica* or the HytA–E hydrogenase in *C. ljungdahlii* [1]. Moreover, *C. autoethanogenum* possesses a functional complex consisting of a NADP ^+^ -specific electron-bifurcating [FeFe]-hydrogenase [73]. Interestingly, while *C. autoethanogenum* reaches indeed higher biomass yields on H_2_ + CO_2_ compared to *A. woodii*, its growth rate is lower [74]. Our analysis suggests that the reduced network-wide thermodynamic driving force (herein quantified via MDF) might be the reason for this behavior. Generally, there are few redox cofactor swaps that occur with high frequency in the found solutions (including the two mentioned above). These key swaps, which concentrate on few reactions (Table 1), are largely responsible for the potential enhancements in the growth rate or/and MDF.

Unexpectedly, we also found solutions in the TCOSA model that use exclusively Fdx and NAD(H) as redox cofactor pools (in combination with appropriate cofactor swaps in NADP(H)-linked redox reactions) and that they can reach (and even slightly outperform) growth rates of the wild-type with only minor reductions in the driving forces. The advantage of such solutions would be that maintaining a third redox cofactor pool (NADP(H)), including the expression of the NFN transhydrogenase, would become obsolete. However, we see two major reasons why a solution with only two redox cofactor pools has not been selected by nature. First, it would require many cofactor swaps in (often NADP(H)-linked) biosynthetic pathways that have been shaped by evolution and are conserved in many different types of organisms. Second, operation with Fdx and NAD(H) could work for chemolithoautotrophic growth but could become a problem for heterotrophic conditions, because H_2_ as a major source for Fdx reduction is then absent. The classical partitioning into an oxidized NAD(H) and a reduced NADP(H) pool is then the optimal choice to maintain high driving forces [52], with NADPH as the main electron donor in biosynthetic reactions. Since our calculation showed that operation of the WLP with NAD(H) and NADP(H) alone is thermodynamically infeasible, the best solution under lithoautotrophic conditions is then to use all three redox cofactor pools at optimal reduction degrees (low for NAD(H), medium for NADP(H), and high for Fdx), which also enhances the robustness and flexibility of the system compared to an operation with two redox cofactors.

A comprehensive analysis of the production capabilities of *A. woodii* revealed that, with formate or H_2_ + CO_2_ as substrates, growth-coupled (acetate-independent) synthesis is feasible only for a few relevant target products when no redox cofactor swaps are allowed. Introduction of 1-2 redox cofactor swaps can in most (but not all) cases enable the production of the target chemical or/and increase the driving force. It again turns out that the cofactor swaps resulting in a NADP^+^-linked hydrogenase and a Fdx- or NAD(P)H-dependent formate dehydrogenase (replacing the HDCR) will be most instrumental in achieving high-potential strain designs.

Overall, we used a rigorous model-driven approach to analyze the metabolism of *A. woodii* under thermodynamic constraints and to compute targets for metabolic engineering optimizing driving forces and product yields when using *A. woodii* as host for the production of chemicals. Our modeling approach directly accounts for external gas (or substrate) concentrations and allows for the estimation of lower bounds for the required H_2_+CO_2_ concentrations in gas fermentations. Many findings are of general relevance for acetogens and our methodology can be easily applied to metabolic models of other acetogenic organisms as well.

## Data and code availability

All models, scripts and data used in this work are publicly available via GitHub (https://github.com/klamt-lab/Modeling_Awoodii) and archived on Zenodo (https://doi.org/10.5281/zenodo.18678845).

## CRediT authorship contribution statement

**Jasmin Bauer:** Data curation, Formal analysis, Investigation, Methodology, Software, Validation, Visualization, Writing – original draft, Writing – review & editing. **Axel von Kamp:** Data curation, Formal analysis, Methodology, Software, Writing – review & editing. **Stefan Pflügl:** Data curation, Funding acquisition, Supervision, Writing – review & editing. **Steffen Klamt:** Conceptualization, Formal analysis, Funding acquisition, Investigation, Methodology, Project administration, Supervision, Writing – original draft, Writing – review & editing.

## Declaration of competing interest

The authors declare that they have no known competing financial interests or personal relationships that could have appeared to influence the work reported in this paper.
